# Anti-Inflammatory and Immunomodulatory Effects of Barberry (*Berberis vulgaris*) and Its Main Compounds

**DOI:** 10.1155/2019/6183965

**Published:** 2019-11-19

**Authors:** Rasool Nasiri Kalmarzi, Seyyed Nima Naleini, Damoon Ashtary-Larky, Ilaria Peluso, Leila Jouybari, Alireza Rafi, Fereshteh Ghorat, Nishteman Heidari, Faezeh Sharifian, Jalal Mardaneh, Paola Aiello, Sobhan Helbi, Wesam Kooti

**Affiliations:** ^1^Lung Diseases & Allergy Research Center, Research Institute for Health Development, Kurdistan University of Medical Sciences, Sanandaj, Iran; ^2^Student Research Committee, Kurdistan University of Medical Sciences, Sanandaj, Iran; ^3^Nutrition and Metabolic Diseases Research Center, Ahvaz Jundishapur University of Medical Sciences, Ahvaz, Iran; ^4^Council for Agricultural Research and Economics (CREA), Research Centre for Food and Nutrition, Via Ardeatina 546, 00178 Rome, Italy; ^5^Nursing Research Center, Golestan University of Medical Sciences, Gorgan, Iran; ^6^Student Research Committee, School of Nursing & Midwifery, Shahid Beheshti University of Medical Sciences, Tehran, Iran; ^7^Traditional and Complementary Medicine Research Center, Sabzevar University of Medical Sciences, Sabzevar, Iran; ^8^Department of Microbiology, School of Medicine & Student Research Committee, Gonabad University of Medical Sciences, Gonabad, Iran; ^9^Department of Physiology and Pharmacology “V. Erspamer”, La Sapienza University of Rome, Rome, Italy; ^10^Student Research Committee, Dezful University of Medical Sciences, Dezful, Iran

## Abstract

*Berberis vulgaris* is a well-known herb in Iran that is widely used as a medicinal plant and a food additive. The aim of this study was to investigate the anti-inflammatory and immunomodulatory effects of Barberry and its main compounds. This narrative review was conducted by searching keywords such as *B*. *vulgaris*, Barberry, immunomodulatory, anti-inflammatory, medicinal herbs, plants, and extract, separately or combined in various databases, such as Web of Sciences, PubMed, and Scopus. According to the inclusion and exclusion criteria, just English language articles, which reported effective whole plants or herbal compounds, were included. 21 articles were reviewed in this study. In the *in vivo* models (mice, rats, and human cells) and in the *in vitro* models (some organ cells such as the spleen, kidney, blood, and brain), *B*. *vulgaris* and its main components showed anti-inflammatory effects in both models. The main mechanisms were the shift of cell immune response to Th2, T reg induction, inhibition of inflammatory cytokines (IL-1, TNF, and IFN-*γ*), and stimulation of IL-4 and IL-10. The induction of apoptosis in APCs and other effector cells was another important mechanism.

## 1. Introduction

Medicinal plants can change the body's physiological or pathological mechanisms to prevent or treat diseases. These plants have been used widely from ancient ages for medical purposes. Nowadays, easy access, low cost, and the prevailing belief in the low side effects have led to a dramatic increase in the use of these plants compared to chemical medications [1, 2]. In Iran, the use of medicinal plants is also significantly increased [3, 4]. Medicinal plants can be used for a variety of purposes, including gastroenterology [5], headache and migraine [6], reproductive disorders [7], diabetes [8, 9], hyperlipidemia [10], stress and depression [11, 12], liver disorders [13, 14], and nervous system disorders [15]. Of course, the use of these drugs also has disadvantages, including deficiencies and lack of experiments for purity, health effects, or teratogenicity [16].

*Berberis vulgaris* is a common herb in Iran (called Barberry) and other countries that widely used it as a medicinal plant and its fruit as a food additive. The scientific classification of *B. vulgaris* is given in Table 1 [17]. This shrub plant with a height of 1 to 3 meters is barbed with yellow wood and ovoid leaves and has hanging yellow flowers that ultimately turn to pulled reddish fruits with a length of 7 to 10 mm and a width of 3 to 5 mm. These fruits come ripening in the end of summer and autumn and are edible. They also have a sour taste and are rich in vitamin C [18–20]. This plant is found in regions such as north of America and central and southern Europe, and south of Asia [21, 22]. The various parts of this plant, including stems, roots, fruits and leaveshave been used in Iran and other countries as traditional medicine [23]. In traditional medicine, this plant is used in several purposes, including cleansing of renal stones and urinary tract diseases and gastrointestinal, liver, and gallbladder diseases as well as a circulatory system stimuli [24–26]. Some traditional uses of Barberry are listed in Table 2.

**Figure 1:**
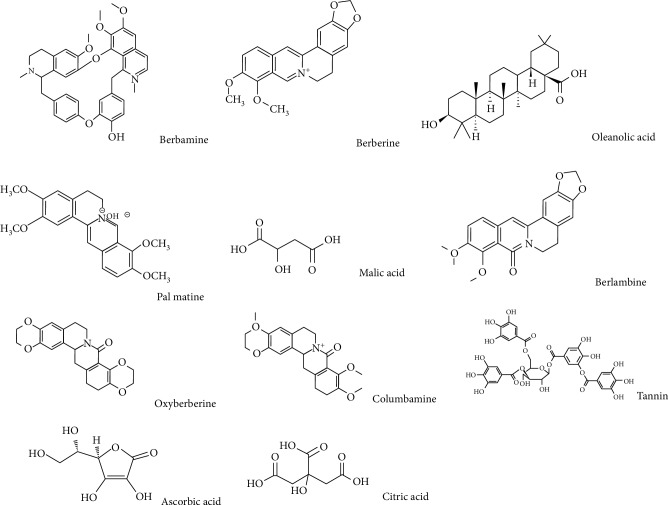
Structure of some important compounds of *B*. *vulgaris*.

About 22 alkaloid compounds have been identified in the roots, the leaves, and the fruits of Barberry [27]. Studies about the chemical components of the extract of this plant show that alkaloids with an isoquinoline core such as protoberberine, berberamine, tetrandrine, chondocurine, and palmatine are among the important contents of Barberry. In the quantitative HPLC (high-performance liquid chromatography) analysis of the principal alkaloids of the root, shell, and stem of *B. vulgaris*, berberine and barbramine were reported 1.24% and 2.5%, respectively. The anti-inflammatory and immunosuppressive properties of these compounds are very important [28–31]. The fruits, flowers, and seeds of this plant contain significant amounts of phenolic compounds (including anthocyanin and carotenoid pigments), pectin, oleoresin, vitamin C, and organic acids such as chelidonic acid, resin, and tannin [18, 20, 32, 33]. The fruits of this plant also have a sour taste and contain dextrose, malic acid, tartaric acid, and citric acid. Barberry extract contains flavonoids such as quercetin, chrysanthamine, hyperoside, dolphinidin-3-O-beta-D-glucoside, pelargonin, petunidin-3-O-beta-D-glucoside, alpha tocopherol, and beta-carotene (Figure 1) [34, 35]. Some of the important compounds found in Barberry are listed in Table 3. For Barberry, many properties are mentioned. In various studies, some pharmacological effects, such as antioxidant and cytoprotective properties [36], inhibition of vascular permeability [37] and epidermal growth factor (EGF) [38], and anticholinergic and antihistaminergic properties [18], for anthocyanins and Barberry fruits are mentioned. The antioxidant activity of Barberry is such that it reduces the survival of cancer cells, and this property is probably due to phenolic compounds and flavonols in the Barberry plant [39, 40].

Studies have shown that alkaloids in this plant increase immunity through T cells [41, 42]. Berberine in the root of Barberry has anticonvulsant, sedative, and diuretic effects [43]. Also, the berberine in Barberry shows the elimination activity of the ONOO(-) and NOO(2)(-) radicals that can contribute to oxidative damage reduction [44]. The extract of this plant is effective in inhibiting activating protein 1 (AP1) of human hepatoma cells [45]. However, the consumption of high levels of alkaloid in this plant (berberine) can cause respiratory paralysis, but death from the high consumption of this plant has not been reported so far. Barberry, by controlling the ACE enzyme (angiotensin converting enzyme) in the brain or by antioxidant property, can reduce the symptoms of Parkinson's disease. Barberry extract inhibits monoamine oxidase A (MAO-A), thus increasing the level of monoamines such as epinephrine and dopamine in the brain, which has an antidepressant effect [28, 43]. Some of the important compounds of this plant are mentioned in Table 3.

Since access to and use of medicinal plants are very easy today, extensive studies on these compounds are important in order to understand their benefits and side effects and can be used to better understanding and explanation of the status of these medications in the community's health. The aim of this study was to investigate the anti-inflammatory and immunomodulatory effects of Barberry and its main compounds.

## 2. Methodology

This narrative review studied the main research performed on anti-inflammatory and immunomodulatory effects of *B*. *vulgaris*. Keywords such as *B*. *vulgaris*, Barberry, immunomodulatory, anti-inflammatory, medicinal herbs, plants, and extract were searched separately or combined in various databases, such as Web of Sciences, PubMed, and Scopus.

According to the inclusion and exclusion criteria, just English language articles, which reported effective whole plants or herbal compounds that performed standard laboratory tests, were included. Unrelated articles that studied other plants were excluded. The titles and abstracts of the studies were evaluated independently by two authors according to the inclusion and exclusion criteria. Data extracted from various articles were included in the study and entered into a check list that included some information: Publication year, authors' name, model type, concentration or dose of extract, and mechanisms of action (if reported).

## 3. Results

21 articles were reviewed in this study. Details of the studies are presented in Table 4. In the following, we will refer to the most important observations of these studies:

In the study of W-C. Lin and J-Y. Lin, anti-inflammatory effects of berberine were investigated in nonobese diabetic (NOD) rats with spontaneously developed type 1 diabetes. The results showed that type 1 diabetes alone causes inflammation in some visceral organs. The administration of berberine reduced spontaneous thymus and spleen inflammation in mice. The secretion of IL-6 by splenocytes in the presence of Con A in NOD mice was significantly higher than that of ICR mice. Berberine administration (especially at high doses of 500 mg/kg BW; 20 g mouse/day) reduced ratios of the Th1 (IL-2)/Th2 (IL-4) cytokines expression in NOD mice splenocytes in the absence/presence of LPS in a preventive way [46]. In the study of Lee et al., the pulmonary inflammation process was simulated by stimulating pulmonary factors. Increased levels of inflammatory cytokines such as IL-1b and TNF-*α* are associated with pulmonary inflammation. This study showed that different inflammatory factors such as lipopolysaccharide, 12-o-tetradecanoylphorbol-13-acetate, hydrogen peroxide, okadaic acid, and ceramide induced the production of IL-1b and TNF-*α* in human pulmonary epithelial cells (A-549), fibroblast (HFL1), and lymphoma cells. However, the result showed that Berberine suppressed inflammatory factors produced due to the production of TNF-*α* and IL-1b in human pulmonary cells [47]. In the study of W-C. Lin and J-Y. Lin, the effects of berberine on the expression of the inflammatory cytokines gene in the initial splenocyte of mice, in the presence and absence of lipopolysaccharide (LPS), were investigated in four experimental models under laboratory conditions. In the initial splenocyte of mice, in the absence and presence of LPS, the anti-inflammatory potential of berberine was reported. A study conducted by Marinova et al. on tubulointerstitial nephritis (TIN) model rats showed that the severity of pathologic damage in TIN rats treated by berberine was significantly lower than that of the control group. In these rats, berberine reduced the number of TCD8+ and TCD4+ lymphocytes. The number of these cells in the peripheral blood is reduced by treatment with berberine and also reduces the infiltrated cells in the kidney [48].

Yan et al. investigated berberine effects on intestinal injury induced by DSS (dextran sulfate sodium) as well as on rat colitis and showed that berberine improved weight loss in rats induced by DSS, as well as the activity of myeloperoxidase, inflammatory scars, and colon injury. In addition, berberine inhibited the production of proinflammatory cytokines such as TNF-*α*, IFN-*γ*, and IL-17 in colon macrophages and epithelial cells in DSS mice [49]. A study by Jiang et al. was conducted to evaluate the protective effects of berberine on neural cells of the brain parenchyma in EAE mice, and the results showed that berberine can reduce MMP9 activity in the brain of EAE mice and, by reducing the expression of MMP9, causes reducing laminin decomposition and thereby preserving brain blood barrier (BBB) that results in a decrease in the number of inflammatory cells infiltrated in the brain and a reduction in the expression of cytokines and chemokines in the brain [50]. In another study, Hu et al. showed that treatment with berberine suppressed the pathological changes caused by the CIA. In the Aziz study, it was found that NO levels in the treated *B*. *vulgaris* cells were 5% lower than the control group (no treatment was applied in this group). In the study of Hu et al., berberine treatment improved EAN symptoms in affected rats [51].

## 4. Discussion

In this study, 21 articles were reviewed. In the *in vivo* models, mice, rat, and human cells, and in the *in vitro* models, some organ cells such as the spleen, kidney, blood, and brain were used. In this section, we mention the most important mechanisms of the effect of *B. vulgaris* and its important compounds, including berberine.

In the study of W-C Lin and J-Y Lin, the anti-inflammatory effects of berberine were attributed to a reduction in TNF-*α*, IL-6, and IL-1b cytokines. The use of berberine in NOD mice was positively correlated with LPS-stimulated IL-10/IL-1b and Con A-stimulated IL-10/TNF-*α*. This effect was particularly higher at high doses (500 mg/kg BW; 20 g mouse/day). In the confirmation of this finding, Lee et al. showed that berberine reduces the inflammatory factors induced by the production of TNF-*α* and IL-1b in human pulmonary cells. Berberine inhibited this mechanism by inhibiting Ik-Ba phosphorylation. Berberine also inhibited the expression of cyclooxygenase 2 (COX2) by the regulation of AP-1 [47]. In the study of W-C Lin and J-Y Lin, change in the expression of Th1 (TNF-*α*) and Th2 (IL-4) cytokines and the shift of Th1/Th2 to Th2 and further increase in the expression of IL-10 and IL-4 were known as the effective processes in the immunomodulatory activity of berberine [46]. Yan et al. showed that berberine improved myeloperoxidase activity, inflammatory scars, and colon damage. In addition, berberine inhibited the production of proinflammatory cytokines such as TNF-*α*, IFN-*γ*, and IL-17 in colon macrophages and epithelial cells in DSS-treated mice [49]. In the study of Ren et al., berbamine altered the response of autoreactive T cells by two ways: on one hand, by inhibiting the nuclear factor of activated T cells (NFAT), it reduced the T cells proliferation; on the other hand, it changed the cytochrome profile of encephalitogenic T cells. By selective inhibition of IFN-*γ* through the Jak/stat pathway and by increasing the SLIM, which is ubiquitin E3 ligase for STAT4, it boosts STAT4 protease degradation and reduces IFN-*γ* production [58]. In study Cui et al., it was found that berberine reduced the differentiation of Th1 and Th17 cells by reducing the expression of lineage markers. The berberine inhibits the differentiation to Th17 by activating ERK1/2. ERK plays this role by decreasing the STAT3 phosphorylation and expressing ROR*γ*t. Berberine also inhibits the differentiation of Th1 by inhibiting the activity of p38 MAPK and JNK. Berberine reduces STAT1 and STAT4 activities by suppressing p38 MAPK and JNK activity and controls the stability of STAT4 through the ubiquitin proteasome pathway [42].

However, the response shift mechanism of Th2 in the study of Marinova et al. was rejected because in their study berberine reduced the number of TCD8+ and TCD4+ lymphocytes in mice, which showed suppression of both Th1 and Th2 cells. The suppression of Th1 response in this study was confirmed by inhibiting the delayed type hypersensitivity in TIN+BB mice and suppression of the Th2 response by decreasing the amount of IL-6 in the studied animals [48].

Another mechanism that is effective in berberine's immunomodulatory effect is the involvement of APCs. By reducing the activity of NF-*κ*B and thus reducing the supply of stimulatory stimulant molecules such as CD80 and CD86 on APCs, berberine reduces antigen delivery performance in these cells. Berberine also suppresses the production of IL-6, IL-12p40, and IL-23p19 in APCs. In confirmation of these findings, Hu et al. showed that berberine can limit the maturity of DCs and reduce their longevity by inducing selective apoptosis. Berberine can lead to oxygen free radical production, activation of caspase-3, and polarization loss in the mitochondrial membrane of DCs, which together result in the apoptosis of these cells [51].

But Jiang et al. presented another mechanism for the anti-inflammatory and immunosuppressive properties of berberine. The purpose of these researchers was to evaluate the protective effects of berberine on neural cells of the brain parenchyma in EAE mice, and they reported that berberine can reduce MMP9 activity in EAE mice, and by decreasing the expression of MMP9, it decreases laminin decomposition and thereby preserves the blood brain barrier, resulting in a reduction in the number of inflammatory cells infiltrated in the brain and reduced expression of inflammatory cytokines in the brain [50].

## 5. Conclusion

It seems that *B*. *vulgaris* and its most important component berberine play their anti-inflammatory and immunomodulatory effect through the shift of cell immune response to Th2, T reg induction, inhibition of inflammatory cytokines (IL-1, TNF, and IFN-*γ*), and stimulation of IL-4 and IL-10. The induction of apoptosis in APCs and other effector cells was another important mechanism. Berberine has showed to have several pharmacological properties, including antimicrobial, anticancer, and immunomodulatory activities. However, hypocholesterolemic and hypoglycaemic activities of berberine have come to light recently. Furthermore, berberine seems to have useful effects on the cardiovascular system, owing to its vase-relaxing and hypotensive activities, as well as the potency of prevention of congestive heart failure, cardiac hypertrophy, and arrhythmia. The experimental and clinical evidence available to date in the literature shows interesting employment prospects of berberine in the treatment of hypercholesterolemia and diabetes. This could open the way not only to a new therapeutic possibility effective in the control of hypercholesterolemia in patients who do not tolerate statins but also to new forms of diabetes therapy and all those situations characterized by evident signs of metabolic syndrome, with possible reduction of a cardiovascular risk. In the end, it is recommended to test and apply these effective compounds in the pharmaceutical industry to control both inflammatory and autoimmune and metabolic diseases.

## Figures and Tables

**Table 1:** Scientific classification of B. *vulgaris.*

Kingdom	Plantae
Order	Ranunculales
Family	Berberidaceae
Genus	Berberis
Species	B. vulgaris

**Table 2:** Some traditional uses of *B. vulgaris*.

System	Effect	Part of the plant	Method of use
Cardiovascular	Antihypertensive	Dried leaves	Injection
	Antiedema	Stem shell	Boiled
	Varicose vein treatment	Root	Boiled

Gastrointestinal	Choleretic	Dried whole plant	Injection
	Cholagogue	Root	Aqueous extract
	Diarrhea	Root	Root
	Bowel movement	Dried root shell	Boiled
	Liver and gallbladder disorders	Dried root shell	Root
	Intestinal ulcers	Root	Root
	Hepatitis	Dried root shell	Boiled

Endocrine	Dysmenorrhea	Fruit Dried root	Aqueous extract
	Menorrhagia	Root	Aqueous extract
	Contraceptive	Root	Aqueous extract

Immune system	Anti-inflammatory	Dried root	Root
	Rheumatoid arthritis	Dried root shell+stem shell	Boiled
	Gout	Flower	Boiled

Central nervous system	Reduce fever	Dried fruit	Aqueous extract
	Sedative	Dried root	Root

Renal	Diuretic	Dried root	Boiled
	Kidney inflammation	Dried root	Root
	Nephritis	Dried root shell	Boiled

**Table 3:** Important compounds of *B. vulgaris.*

Compound	Nature	Part of the plant
Acanthine	Alkaloid	Root, root shell, stem shell, leaves, sprout
Berbamine	Alkaloid	Root, stem shell, fruit
Berberine	Alkaloid	Root, stem, fruit
Berlambine	Alkaloid	Root
Bervulcine	Alkaloid	Root
Columbamine	Alkaloid	Root, stem shell
Tannin	Tannin	Fruit
Pectin	Carbohydrate	Fruit
Delphinidin-3-o-beta-d-glucoside	Flavonid	Leaves
Ascorbic acid	Vitamin	Fruit, leaves
Palmatine	Alkaloid	Root, stem shell, fruit
Quercetin	Flavonid	Leaves
Petunidin-3-o-beta-d-glucoside	Flavonid	Fruit
Malic acid	Alkane to c4	Fruit
Vitamin K	Vitamin	Leaves
Alpha-tocopherol	Oxygen	Leaves

**Table 4:** Some of the information about the anti-inflammatory and immunomodulatory effects of *B. vulgaris.*

Part of plant	Fraction or isolated compounds	Model	Disease model	Tissue	Effect	Dose	Refs.
Whole plant	Berberine	*In vivo*	Nonobese diabetic (NOD) mice	Spleen, liver, or kidney	↓ Th1/Th2 cytokines	50, 150, 500 mg	[52]
Whole plant	Berberine	*In vitro*	Normal	Human lung epithelial cells and fibroblasts	↓ Inflammatory cytokine	5 *μ*M	[47]
Whole plant	Berberine	*In vitro*	Normal	Primary splenocytes in mice	↓ TNF-*α*, IL-2, IL-4, IL-10	0.8, 1.6, 3.3 *μ*M	[46]
Root	Berberine	*In vitro*	Arthritis and *Candida albicans* infection	Spleen	↑ Joint inflammation, *C. albicans* infection not affected	5, 10 mg	[41]
Root	Berberine	*In vivo*	Normal	NM	Antinociceptive and anti-inflammatory	20, 40, 80, 160 mg/kg	[53]
Whole plant	Berberine	*In vivo*, *in vitro*	Normal	Splenic macrophages and dendritic cell	↓ IL-4, ↑ IL-12, ↑ IFN-*γ*	100 *μ*g	[54]
Fruit	Berberine	*In vivo*	Acute and chronic inflammation in male rat	Groin border of rats	↑ Light absorption of peritoneal fluid, ↓ chronic inflammation, ↓ chronic pain	75, 150, 300 mg/kg	[55]
Root	Berberine, oxyacanthine	*In vivo and in vitro*	Paw edema in mice	Splenocytes	↓ Delayed type hypersensitivity	20, 100, 200	[28]
Fruit	Berberine	*In vitro*	Normal	Human peripheral lymphocytes	↓ CD69	25, 50, 100 *μ*mol/L	[56]
NM	Berberine	*In vivo*, *in vitro*	Normal	Splenic dendritic cell	↑ Apoptosis in DC	1, 50 mg/kg	[51]
Root	Berberine	*In vitro*	Immunodeficiency	Splenic dendritic cell and splenocytes	↑ IL-12, ↑ IFN-*γ*, ↑ CD11c	100 *μ*g/mL	[57]
NM	Berbamine	*In vivo*, *in vitro*	Autoimmune encephalomyelitis	Splenocyte	↓ IFN-*γ*	50 mg/kg	[58]
NM	Berberine	*In vivo*	Autoimmune encephalomyelitis	Spinal cord	↓ Th17, ↓ NF-*κ*B	200 mg/kg	[59]
NM	Berberine	*In vivo*	Autoimmune neuritis	Lymph node mononuclear cells	↓ TNF-*α*, ↓ IL-10	20 and 130 mg/kg	[60]
NM	Berberine	*In vivo*	Type 1 diabetic mice	Splenocyte	↓ STAT1, ↓ STAT4, ↓ Th17	200 mg/kg	[42]
NM	Berberine	*In vivo*	Normal	Splenocyte	↑ IL-12p40	1 *μ*g/mL	[61]
NM	Berberine	*In vivo*	Autoimmune tubulointerstitial nephritis	Kidney	↓ CD3+, ↓ CD4+, ↓ CD8+, ↓ TIN	10 mg/kg	[48]
NM	Berberine	*In vivo*	Dextran sulfate sodium- (DSS-) induced intestinal injury and colitis in mice	Colon	↓ TNF, ↓ IFN-*γ*, ↓ KC, ↓ IL-17	100 mg/kg	[49]
NM	Berberine	*In vivo*	Autoimmune encephalomyelitis	Brain	↓ TUNEL-positive neuronal cells, ↓ gelatinase activity, ↓ laminin degradation	30 mg/kg	[50]
Root	Berberine	*In vivo*	Toxoplasma gondii infection	Peritoneal fluid	Treatment of parasitic infection such as acute toxoplasmosis	1, 2 g/kg	[62]
Fruit	Berberine	*In vitro*	Bacterial vaginosis	Vagina	Treatment of bacterial vaginosis	0.75%, 2%, 5%	[63]

NM: not mentioned.
